# Rapid Response to SARS-CoV-2 in Aotearoa New Zealand: Implementation of a Diagnostic Test and Characterization of the First COVID-19 Cases in the South Island

**DOI:** 10.3390/v13112222

**Published:** 2021-11-04

**Authors:** Blair Lawley, Jenny Grant, Rhodri Harfoot, Jackson M. Treece, Robert Day, Leonor C. Hernández, Jo-Ann L. Stanton, James E. Ussher, Miguel E. Quiñones-Mateu

**Affiliations:** 1 Department of Microbiology & Immunology, School of Biomedical Sciences, University of Otago, 9054 Dunedin, New Zealand; blair.lawley@otago.ac.nz (B.L.); rhodri.harfoot@otago.ac.nz (R.H.); leonor.hernandez@otago.ac.nz (L.C.H.); 2 Southern Community Laboratories, Dunedin Hospital, 9054 Dunedin, New Zealand; jenny.grant@sclabs.co.nz; 3 Department of Anatomy, School of Biomedical Sciences, University of Otago, 9054 Dunedin, New Zealand; jackson.treece@otago.ac.nz (J.M.T.); jo.stanton@otago.ac.nz (J.-A.L.S.); 4 Department of Biochemistry, School of Biomedical Sciences, University of Otago, 9054 Dunedin, New Zealand; robert.day@otago.ac.nz; 5 Webster Centre for Infectious Diseases, University of Otago, 9054 Dunedin, New Zealand

**Keywords:** SARS-CoV-2, COVID-19, diagnostic test, whole-genome sequencing, New Zealand

## Abstract

It has been 20 months since we first heard of SARS-CoV-2, the novel coronavirus detected in the Hubei province, China, in December 2019, responsible for the ongoing COVID-19 pandemic. Since then, a myriad of studies aimed at understanding and controlling SARS-CoV-2 have been published at a pace that has outshined the original effort to combat HIV during the beginning of the AIDS epidemic. This massive response started by developing strategies to not only diagnose individual SARS-CoV-2 infections but to monitor the transmission, evolution, and global spread of this new virus. We currently have hundreds of commercial diagnostic tests; however, that was not the case in early 2020, when just a handful of protocols were available, and few whole-genome SARS-CoV-2 sequences had been described. It was mid-January 2020 when several District Health Boards across New Zealand started planning the implementation of diagnostic testing for this emerging virus. Here, we describe our experience implementing a molecular test to detect SARS-CoV-2 infection, adapting the RT-qPCR assay to be used in a random-access platform (Hologic Panther Fusion^®^ System) in a clinical laboratory, and characterizing the first whole-genome SARS-CoV-2 sequences obtained in the South Island, right at the beginning of the SARS-CoV-2 outbreak in New Zealand. We expect that this work will help us and others prepare for the unequivocal risk of similar viral outbreaks in the future.

## 1. Introduction

SARS-CoV-2, the causative agent of COVID-19, emerged in Wuhan (Hubei province, China) in December 2019 [1]. The virus quickly spread to several countries, prompting the World Health Organization to declare a Public Health Emergency of International Concern on 30 January 2020 and a COVID-19 Pandemic on 11 March 2020 (https://www.who.int/, accessed on 7 September 2021). Eighteen months later (as of 31 August 2021), the virus has been responsible for over 217 million COVID-19 cases and more than 4.5 million deaths worldwide [2].

Following the first description of the viral disease in Wuhan, and the imminent possibility of its expansion outside China [1,3,4,5,6,7], many researchers, clinicians, and public health authorities across the world were rushing to find the best way to respond to the virus outbreak. Most plans included the development and implementation of diagnostic methods, with the caveat that access to reagents (e.g., SARS-CoV-2 genetic material to be used as control) in countries with no COVID-19 cases was limited. It was clear from the beginning that diagnosis of SARS-CoV-2 infection would rely upon the detection of viral genomic RNA in samples from the upper respiratory tract, using molecular methodologies, such as reverse transcription-polymerase chain reaction (RT-PCR). Numerous RT-PCR assays were quickly developed in academic and clinical laboratories [8,9,10,11], using primers and probes initially designed according to whole-genome SARS-CoV-2 sequences published in January 2020 [1]. These initial tests targeted a variety of SARS-CoV-2 genes (e.g., E, envelope; N, nucleocapsid; RdRp, RNA-dependent RNA polymerase), showing excellent analytical sensitivity, often assessed with synthetic genes, but diverse clinical sensitivity when used on patient-derived specimens [11,12,13,14]. Since then, a multitude of RT-PCR assays based on a variety of methodologies and platforms, many of them approved by different regulatory agencies across the world, are being used to monitor and try to control the COVID-19 pandemic [11,14].

The first case of COVID-19 in New Zealand was identified on 28 February 2020, and border restrictions, compulsory self-isolation following travel, and cruise ship restrictions were introduced on 16 March 2020, when a total of 16 COVID-19 cases had been identified, all imported [15,16]. The country went into full lockdown on 25 March 2020, when there were close to 300 confirmed COVID-19 cases and clear circulation of the virus in the community [17]. These rapid measures eliminated the spread of SARS-CoV-2 in New Zealand for over a year, apart from a couple of brief, rapidly controlled border-related incursions, limiting the infections to a reduced number of imported COVID-19 cases restricted to quarantine facilities [18]. The country returned to Alert 4, lockdown on 18 August 2021 following the detection of the Delta SARS-CoV-2 variant circulating in the community. As of 25 August 2021, New Zealand had a total of 3,159 COVID-19 cases and 26 deaths (https://nzcoviddashboard.esr.cri.nz/#!/, accessed on 25 August 2021).

Here we describe our experience reacting and planning a swift response to the potential threat of an emergent virus in New Zealand. Our initial conversations in mid-January 2020 led to the implementation of a molecular diagnostic test, based on the original protocol described by Corman et al. [8], on a random-access platform (Hologic Panther Fusion^®^ System) less than two months later, just as SARS-CoV-2 was arriving in the country. We also describe the characterization of the first whole-genome SARS-CoV-2 sequences in the South Island, following the emerging SARS-CoV-2 outbreak in New Zealand.

## 2. Materials and Methods

### 2.1. SARS-CoV-2 Synthetic Controls

**Figure 1:**
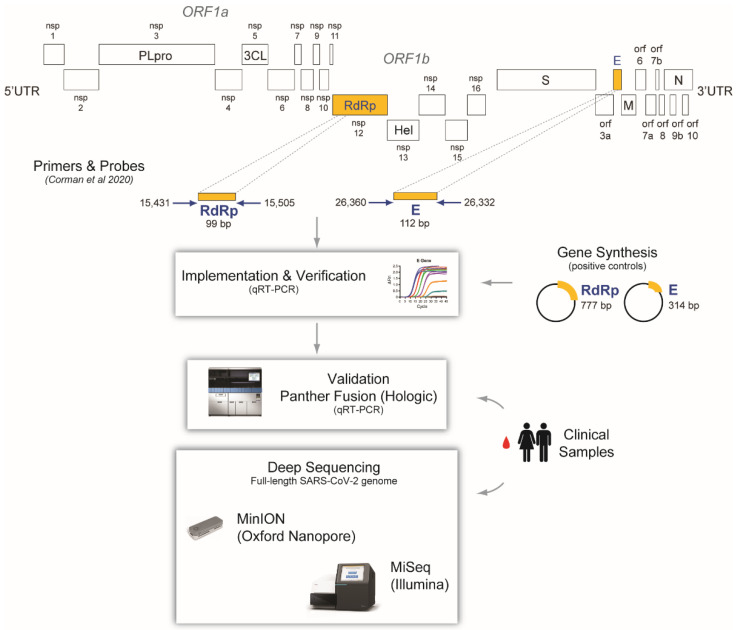
Overview of the implementation and validation of the RT-qPCR SARS-CoV-2 diagnostic test and characterization of the first COVID-19 cases in the South Island, New Zealand. Primers and probes, targeting small regions in the RNA-dependent RNA polymerase (RdRp) and envelope (E) SARS-CoV-2 genes were obtained from Corman et al. [8]. Two fragments corresponding to the RdRp (777 bp, position 14,902 to 14,946; Wuhan-Hu-1 NC045512) and the E (314 bp, position 26,245 to 26,265; Wuhan-Hu-1 NC045512) SARS-CoV-2 genes were synthesized and cloned into the pBluescript II KS(+) plasmid (GenScript Biotech, Singapore) to be used as controls. Primers, probes, plasmid controls, and clinical samples were used to implement and validate the molecular test in the Hologic Panther Fusion^®^ System. The whole genome of the viruses corresponding to the first two SARS-CoV-2 infections identified in the South Island was characterized using both MinION (Oxford Nanopore Technologies, Oxford, UK) and MiSeq (Illumina, San Diego, CA, USA) deep sequencing platforms. 5′UTR, 5′ untranslated region; nsp1 to 16, non-structural proteins 1 to 16; ORF1a to 10, open reading frame 1a to 10; PLpro, papain-like protease; 3CL, 3C-like protease; RdRp, RNA-dependent RNA polymerase; Hel, helicase; S, spike; E, envelope; M, membrane; N, nucleocapsid; 3′UTR, 3′ untranslated region.

The complete genome sequence of SARS-CoV-2 Wuhan-Hu-1 (MN908947) was downloaded from NCBI (https://www.ncbi.nlm.nih.gov/nuccore/MN908947.3, accessed on 15 March 2020) and used to synthesize two SARS-CoV-2 gene fragments as controls. Briefly, gene synthesis was used to generate two sequences corresponding to the envelope (E) and RNA-dependent RNA polymerase (RdRp) SARS-CoV-2 genes (314 bp, position 26,245 to 26,265 and 777 bp, position 14,902 to 14,946, Wuhan-Hu-1 NC045512), respectively, and cloned into the pBluescript II KS(+) plasmid (GenScript Biotech, Singapore) (Figure 1).

### 2.2. In Vitro Transcription of SARS-CoV-2 RNA

The pBluescript II KS(+) DNA plasmids containing the E (314 bp) and RdRp (777 bp) fragments were linearized with *Bam*HI and transcribed using the MAXIscript™ T7 Transcription Kit (ThermoFisher Scientific, Waltham, MA, USA). The RNA transcripts were treated with Turbo™ DNase (ThermoFisher Scientific), purified with a RNeasy mini kit (QIAGEN, Valencia, CA, USA), and quantified using a Qubit RNA HS Assay kit (ThermoFisher Scientific). The RNA transcripts were used as positive controls in the implementation of the real-time reverse transcription-quantitative PCR (RT-qPCR) assay.

### 2.3. In-House SARS-CoV-2 RT-qPCR Assay

We adapted a protocol originally described by Corman et al. [8]. Briefly, a set of primers and probe were used for two SARS-CoV-2 genomic regions, i.e., E-Sarbeco_F1 (5′-ACAGGTACGTTAATAGTTAATAGCGT-3′, position 26,269), E-Sarbeco_R2, (5′-ATATTGCAGCAGTACGCACACA-3′, position 26,360), and E-Sarbeco-P1 (5′-FAM-ACACTAGCCATCCTTACTGCGCTTCG-BBQ-3′, position 26,332) for the E gene, and RdRp_SARSr-F2 (5′-GTGARATGGTCATGTGTGGCGG-3′, position 15,431), RdRp_SARSr-R1 (5′-CARATGTTAAASACACTATTAGCATA-3′, position 15,505), and RdRp_SARSr-P2 (5′-FAM-CAGGTGGAACCTCATCAGGAGATGC-BBQ-3′, position 15,470) for the RdRp gene. RNA samples (1.5 µL) were reverse transcribed and amplified in 10 µL reactions containing 0.2 µL of each specific forward and reverse primers (20 pM/µL) and probe (10 pM/µL), 5 µL of qScript^®^ XLT One-Step RT-qPCR ToughMix^®^, Low ROX™ (Quantabio, Gaithersburg, MD, USA), and 2.9 µL of UltraPure™ DEPC-Treated Water (ThermoFisher Scientific). The RT-qPCR assay was performed in the QuantStudio™ 6 Flex Real-Time PCR system (ThermoFisher Scientific) using the following cycling conditions: one cycle at 50 °C for 10 min; one cycle at 95 °C for 3 min; and 45 cycles of 95 °C for 5 s, 58° C for 7 s, and 60 °C for 23 s in 384-well plates.

### 2.4. Adapting the SARS-CoV-2 RT-qPCR Assay to the Hologic Panther Fusion^®^ System

The in-house SARS-CoV-2 RTf-qPCR assay was adapted to use the Open Access function on the Panther Fusion^®^ System (Hologic, Inc, Marlborough, MA, USA), a random-access platform that combines extraction of genetic material and real-time RT-PCR [19]. For that, 500 µL of a patient-derived or laboratory-generated sample was transferred into a specimen lysis tube containing 710 µL of lysis buffer (Hologic^®^), then loaded onto the Panther Fusion^®^ System. The instrument removed 360 µL of the mixture for nucleic acid extraction using the Panther Fusion Extraction Reagent-S (Hologic^®^), which contained an internal control, based on captured oligonucleotides. Extracted nucleic acid was eluted in a final volume of 50 µL, and a small aliquot (5 µL) was RT-PCR amplified in 25-µL reactions containing the primer–probe mix (PPM) solution in the Open Access RNA/DNA Enzyme Cartridge (Hologic^®^). The PPM solution contained KCl (62.5 mM), MgCl_2_ (3.75 mM), Tris (10 mM), and SARS-CoV-2 gene fragment-specific forward and reverse primers for either the “E” assay (E-Sarbeco_F1 and E-Sarbeco_R2, 0.5 µM) or the “RdRp” assay (RdRp_SARSr-F2, 0.75 µM, and RdRp_SARSr-R1, 1 µM) and the appropriate probe (E-Sarbeco-P1, 0.25 µM or RdRp_SARSr-P2, 0.125 µM, respectively). Thermocycling conditions for both E and RdRp assays consisted of one cycle at 50 °C for 8 min and 5 s; one cycle at 95 °C for 1 min; and 44 cycles of 95 °C for 5 s, 58 °C for 7 s, and 60 °C for 14 s.

### 2.5. Clinical Specimens

Nasopharyngeal (NP) swabs were collected from individuals with clinical signs or symptoms consistent with COVID-19 in the South Island, New Zealand, between March and April 2020. A sterile swab made from Dacron, rayon, or nylon was used for each sample collection, then placed into a 3-mL universal transport medium (UTM, various manufacturers). NP samples were transported to the laboratory at room temperature (no more than 30 min) and tested as soon as possible after collection; otherwise, samples were stored at 2 to 8 °C for up to 72 h. After testing, samples were aliquoted and stored at −80 °C. NP samples and basic demographic information were collected with the understanding and written consent of each participant. The study was reviewed and approved by the University of Otago Human Ethics Committee (H21/134).

### 2.6. Whole Genome Sequencing of SARS-CoV-2

Aliquots of NP samples originally stored at −80 °C were used to extract total RNA using a QIAamp Viral RNA Mini Kit (QIAGEN), eluted in 20 µL of DNase/RNase-free water, and used to synthesize complementary DNA (cDNA). Briefly, RNA samples (4 µL) were incubated with 1 µL of DNAse I (1 U/µL, Promega, Madison, WI, USA) at 37 °C for 30 min. Then the reaction was stopped by adding 1 µL of DNase Stop Solution (Promega) and incubated at 65 °C for 10 min. cDNA was randomly synthesized as follows: RNA samples were incubated with adapter appended random nonamer primers (5′-GCCGACTAATGCGTAGTCNNNNNNNNN-3′, 50 pM, IDT) at 85 °C for 2 min and room temperature for 20 min. This mixture was then amplified in 10 µL reactions containing 0.5 µL of SuperScript™ III (200 U/µL, ThermoFisher Scientific), 2 µL of 5× First-Strand Buffer (250 mM Tris-HCl, 375 mM KCl, 15 mM MgCl_2_, ThermoFisher Scientific), 0.5 µL of dNTPs (0.5 µL), 1 µL of DTT (0.1 mM), and 0.5 µL of RiboLock RNase inhibitor (40 U/µL, ThermoFisher Scientific), then incubated at 25 °C for 10 min, 50 °C for 60 min, 95 °C for 2 min, and 4 °C for 2 min. Second strand DNA was generated using Sequenase™ Version 2.0 DNA Polymerase (ThermoFisher Scientific) by ramping the temperature from 4 °C to 37 °C in a period of 8 min, then incubating at 37 °C for 60 min and 94 °C for 2 min. cDNA/second strand products were amplified in 25-µL reactions containing 0.5 µL of DreamTaq DNA polymerase (5 U/µL, ThermoFisher Scientific), 2.5 µL of DreamTaq 10× Buffer, 0.5 µL of dNTPs (10 mM), and 0.5 µL of adapter sequence (100 pM/µL, 5′-GCCGACTAATGCGTAGTC-3′) with the following cycling conditions: one cycle at 95 °C for 2 min, 30 cycles of 95 °C for 30 s, 55 °C for 30 s, and 72 °C for 90 s, and one cycle at 72 °C for 5 min. PCR products were purified (QIAquick PCR Purification kit, Qiagen), the concentration of double-stranded cDNA quantified (Qubit 2.0, ThermoFisher Scientific) and stored at −80 °C until further use.

Whole-genome sequencing was performed with a combination of Oxford Nanopore Technologies (Oxford, UK) and Illumina (San Diego, CA, USA) platforms.

#### 2.6.1. MinION, Oxford Nanopore Technologies

A pool of purified PCR products, averaging ~700 bp (Qubit™ dsDNA HS assay kit, ThermoFisher Scientific), was diluted to a starting concentration of 200 fmol following the recommended protocol (NBA_9093_v109_12Nov2019, Oxford Nanopore Technologies). Briefly, the amplicons were processed using the NEBNext^®^ Ultra™ II End Repair/dA-Tailing Module (New England BioLabs, Ipswich, MA, USA) to give repaired DNA with 5′ phosphorylated, 3′ dA-tailed ends. Barcode adapters (Native Barcoding Expansion 1-12 EXP-NBD104 kit, Oxford Nanopore Technologies) were added to the repaired amplicons using the Ligation Sequencing Kit (SQK-LSK109, Oxford Nanopore Technologies), followed by DNA purification (Agencourt AMPure XP, Beckman Coulter, Brea, CA, USA). Individually barcoded DNA samples were quantified (Qubit 2.0, ThermoFisher Scientific) and mixed at equimolar concentrations. The pooled DNA library was loaded onto a primed MinION R9.4.1 flow cell (Oxford Nanopore Technologies) and run for 15 h. MinKNOW Core 3.6.5 (Oxford Nanopore Technologies) was run on the MinIT device (Oxford Nanopore Technologies) for real-time analysis, basecalling, barcode demultiplexing, and to generate sample-specific fastq files.

#### 2.6.2. MiSeq, Illumina

Dual indices (barcodes) and Illumina sequencing adapters were added to the amplicons (1 ng) by indexing PCR products using the Nextera XT DNA Library Preparation kit (Illumina), followed by DNA purification (Agencourt AMPure XP, Beckman Coulter). Individual barcoded DNA samples were then quantified (Qubit 2.0, ThermoFisher Scientific), normalized to 4 nM, and pooled. The paired-end multiplexed library (two samples plus 5% PhiX as internal control) was diluted to 20 pM and denatured with NaOH prior to sequencing on the MiSeq system (Illumina) using the MiSeq Reagent Kit v2 Nano 300 cycle (2 × 150 bp, Illumina). Indexed reads were demultiplexed and filtered to remove short reads (<80 bp), generating sample-specific fastq files using BaseSpace (Illumina).

Finally, fastq files obtained from both MinION (Oxford Nanopore Technologies) and MiSeq (Illumina) were analyzed using a combination of software packages to characterize the whole genome SARS-CoV-2 sequences: (i) GISAID (https://www.gisaid.org/, accessed on 15 April 2020), (ii) DRAGEN Bio-IT Platform (Illumina), (iii) Genome Detective Virus Tool (https://www.genomedetective.com/, accessed on 15 April 2020), and (iv) IDSEQ (https://idseq.net/, accessed on 15 April 2020).

### 2.7. Phylogenetic Analysis

A small subset of whole-genome sequences was downloaded from the GISAID database (https://www.gisaid.org/, accessed on 7 May 2020) [20] in June 2020 to assess the phylogeny of the two SARS-CoV-2 sequences described in this study, i.e., 28 SARS-like beta coronaviruses and 70 contemporary SARS-CoV-2 sequences from different lineages. Whole-genome SARS-CoV-2 consensus sequences, corresponding to each patient-derived NP sample, were aligned using ClustalW [21] and their phylogeny reconstructed using the Maximum Likelihood model with bootstrap as the variance estimation method (1000 replicates) as implemented within MEGA 6.1 [22].

### 2.8. Statistical Analysis

Descriptive results are expressed as median values and confidence intervals. Pearson’s correlation coefficient was used to determine the strength of association between categorical variables. The cutoff level for significance was set at 0.05 (*p* < 0.05). All statistical analyses were performed using GraphPad Prism v.9.2.0 (GraphPad Software, San Diego, CA, USA) unless otherwise specified. Whole-genome SARS-CoV-2 sequences obtained by deep sequencing in this study have been submitted to GISAID (https://www.gisaid.org/, accessed on 15 August 2020) under the following accession numbers: EPI_ISL_417211 (NZ/Queenstown/01 or hCoV-19/NewZealand/CoV001/2020) and EPI_ISL_417212 (NZ/Dunedin/01 or hCoV-19/NewZealand/CoV002/2020).

## 3. Results

### 3.1. 23 January to 10 March 2020: Implementing a RT-qPCR Assay to Detect SARS-CoV-2

**Figure 2:**
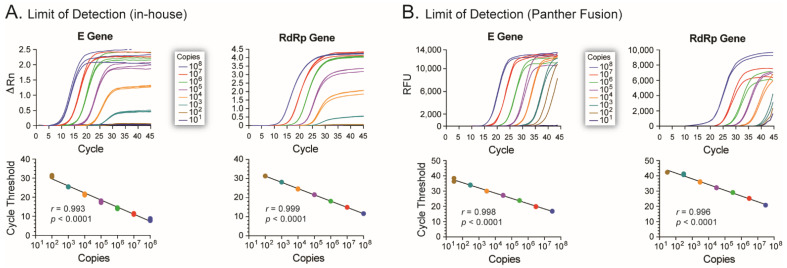
Analytical sensitivity of the RT-qPCR COVID-19 diagnostic test. The ability of the in-house (**A**) and Panther Fusion^®^ System (**B**) RT-qPCR assays to detect SARS-CoV-2 was evaluated using serial dilutions of RNA in vitro transcribed from the RdRp and E plasmid controls. Real-time PCR and calibration curves are indicated for both SARS-CoV-2 genes in both versions of the RT-qPCR assay. Rn, magnitude of signal (Rn value of the reaction with template minus the Rn value of the unreacted sample, without template); Ct, cycle threshold; Copies, number of in vitro-transcribed SARS-CoV-2 RNA copies; RFU, relative fluorescence units. Linear dynamics, regression (*r*) and two-tailed *P* (*p*) values are indicated.

By the third week of January 2020, it was evident that the 2019 novel coronavirus (2019-nCoV, the original name of SARS-CoV-2) [1], was spreading outside Wuhan, China [3,23,24]. On 23 January 2020, we decided to start working on a molecular diagnostic test to detect 2019-nCoV, to be ready before this new virus found its way to New Zealand. At that point, the Drosten group in Germany had published an excellent description of a real-time RT-PCR to detect 2019-nCoV [8], and we started working on implementing this assay in our laboratory. Like many other groups during the early phase of the COVID-19 pandemic, we did not have access to SARS-CoV-2 samples to be used as controls. For that, we ordered two plasmids containing fragments of the SARS-CoV-2 E and RdRp genes, two of the regions used in the Corman et al. assay [8] (Figure 1) and used them to characterize and evaluate the analytical sensitivity of the in-house RT-qPCR assay. Using in vitro-transcribed RNA as standard, the technical limit of detection (LOD) was 100 RNA copies/reaction for both the E and RdRp genes (Figure 2A).

**Figure 3:**
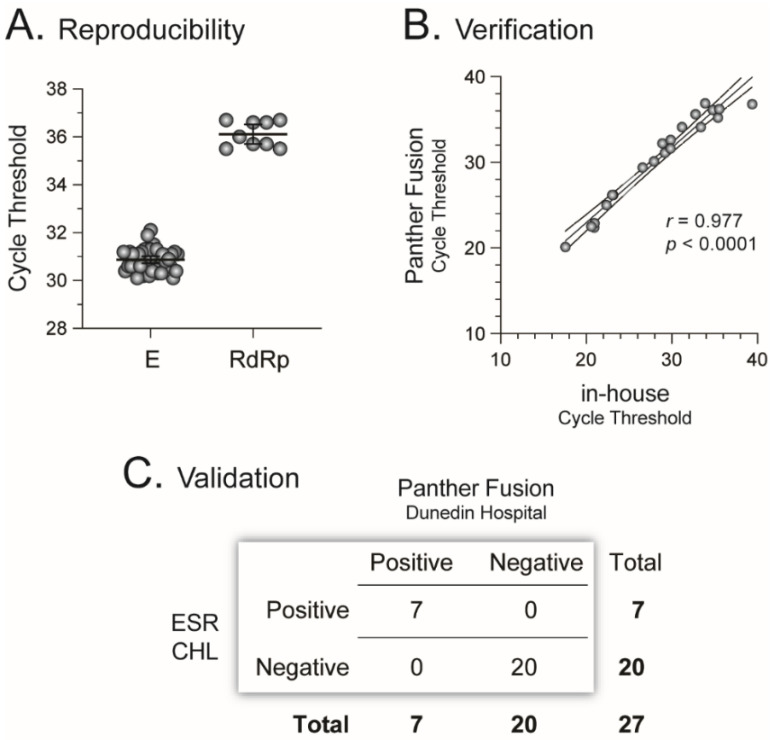
Verification and validation of the RT-qPCR COVID-19 diagnostic assay in the Panther Fusion^®^ System. (**A**) Assay reproducibility was evaluated using in vitro transcribed E or RdRp RNA standards (10^5^ copies) to spike 500 µL of fresh universal transport medium. Each in vitro transcribed RNA was evaluated in multiple replicates (E, *n* = 43 and RdRp, *n* = 10). (**B**) Twenty-one nasopharyngeal (NP) swabs from COVID-19 patients were tested with both the in-house and Panther Fusion^®^ versions of the RT-qPCR assay. r, correlation coefficient and *p*, two-tailed *p* value are indicated. (**C**) Concordance between the Panther Fusion^®^ System assay and COVID-19 diagnostic tests performed in other clinical laboratories in New Zealand (CHL, Canterbury Health Laboratories in Christchurch and ESR, Institute of Environmental Science and Research in Wellington). The concordance between the assays was calculated as follows: number of tests with a concordant result (e.g., positive–positive or negative–negative) with both assays, divided by the total number of determinations (i.e., 27), multiplied by 100.

Based on the results obtained in the research laboratory, we transferred and implemented the RT-qPCR assay in our clinical laboratory (Southern Community Laboratories, Dunedin Hospital, Dunedin, New Zealand). There, we used the Open Access function on the Panther Fusion^®^ System (Hologic, Inc., Marlborough, MA, USA) to characterize and verify the RT-qPCR assay in this fully automated diagnostic platform. Following a series of test runs, we used the in vitro-transcribed RNA standards to show that the RT-qPCR assay in the Panther Fusion^®^ System was able to accurately detect as low as 30 and 300 RNA copies/reaction of the E and RdRp genes, respectively (Figure 2B). The ability to detect only SARS-CoV-2, and not any other pathogen, was initially evaluated using 23 NP samples from individuals positive and/or negative to other respiratory viruses, such as Respiratory Syncytial Virus, Influenza A and B viruses, Human Metapneumovirus, etc., showing 100% specificity for SARS-CoV-2. Next, we used 10^5^ copies of the in vitro transcribed RNA standards to spike 500 µL of fresh UTM to evaluate the reproducibility of the RT-qPCR assay in the Panther Fusion^®^ System. We obtained similar cycle threshold (Ct) values when testing the same samples multiple times, i.e., median Ct values of 31 (95% CI 30.7–31.0, coefficient of variation 1.51%) and 36 (95% CI 35.7–36.5, coefficient of variation 1.47%) for the E and RdRp genes, respectively (Figure 3A).

### 3.2. 13 to 27 March 2020: Validating the RT-qPCR Assay in the Panther Fusion^®^ System to Detect SARS-CoV-2

It was 28 February 2020 when the first case of COVID-19 was identified in New Zealand: an individual returned to Auckland (North Island) from overseas two days before seeking medical attention. Less than two weeks later, during the week of 13 March 2020, the first three cases of COVID-19 were identified in the South Island, one in Queenstown and two in Dunedin. This was the same week that our assay was fully deployed at Southern Community Laboratories. Numerous NP samples started to be tested across the country, including using our newly implemented RT-qPCR assay in the Panther Fusion^®^ System. Therefore, it was not long before we were able to accumulate a number of NP samples positive for SARS-CoV-2. We extracted viral RNA (QIAamp Viral RNA mini Kit, QIAGEN) from 21 patient-derived samples originally analyzed in the Panther Fusion^®^ System (clinical laboratory), then assessed them using the original in-house RT-qPCR assay in our research laboratory, showing a good correlation between both versions of the same diagnostic test (*r* = 0.977, *p* < 0.0001, Pearson’s coefficient correlation, Figure 3B). Importantly, after the first week of performing the RT-qPCR assay in our clinical laboratory, 27 of the tested NP samples were sent for confirmation to other clinical laboratories in the country, i.e., Canterbury Health Laboratories (CHL) in Christchurch and the Institute of Environmental Science and Research (ESR) in Wellington, showing a 100% overall concordance between our assay and the tests performed in the other clinical laboratories (Figure 3C).

### 3.3. 13 March to 10 April 2020: Using the RT-qPCR Assay in the Panther Fusion^®^ System to Diagnose COVID-19 in the South Island, New Zealand

**Figure 4:**
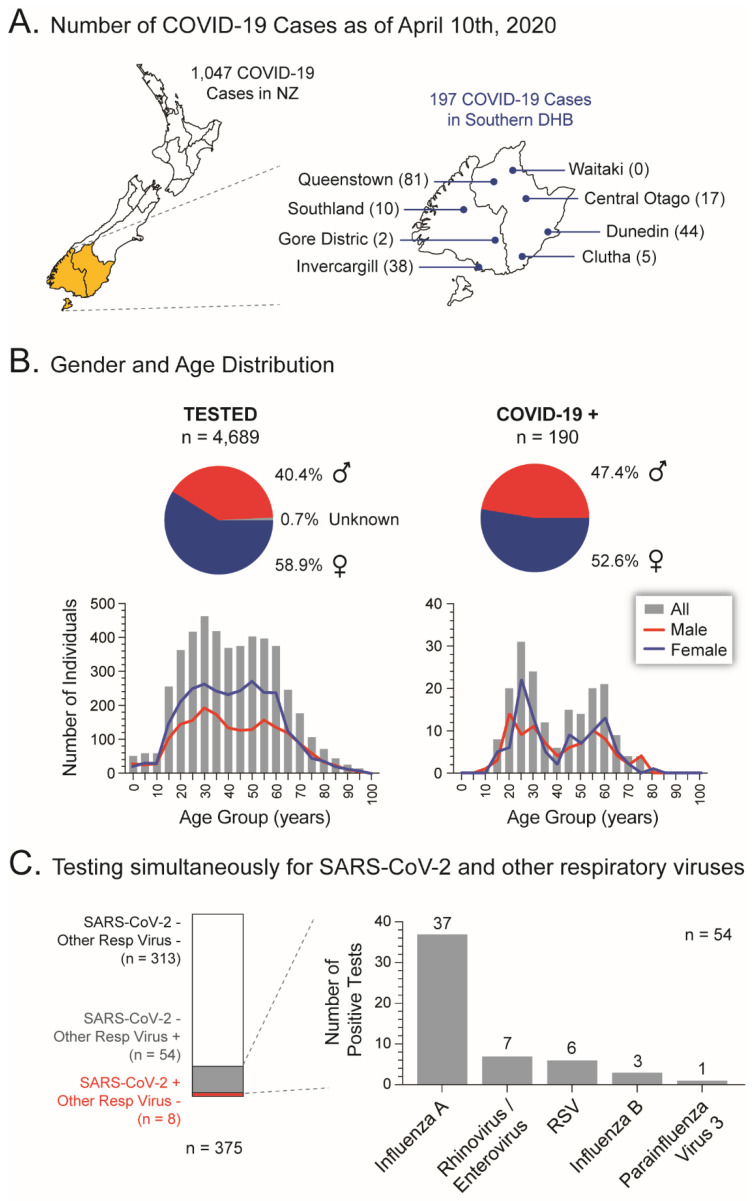
Using the RT-qPCR assay in the Panther Fusion^®^ System to diagnose COVID-19 in the South Island, New Zealand. (**A**) Total number of COVID-19 cases in New Zealand and the Southern DHB during the first month of the SARS-CoV-2 pandemic in the country. (**B**) Gender and age distribution of the total number of individuals tested, as well as COVID-19 cases, in the Southern DHB during the first four weeks of the SARS-CoV-2 outbreak in New Zealand. (**C**) Identifying multiple respiratory virus infections. Co-infections with other respiratory viruses in individuals positive for SARS-CoV-2 were not detected. RSV, Respiratory Syncytial Virus.

During the first four weeks of the SARS-CoV-2 pandemic in New Zealand, a total of 1,047 COVID-19 cases were identified, 197 in the Southern District Health Board (DHB) region, mainly in Queenstown (41.1%), Dunedin (22.3%), and Invercargill (19.3%) (https://nzcoviddashboard.esr.cri.nz/#!/, accessed on 20 June 2020) (Figure 4A). Most of the COVID-19 cases in the Southern DHB (*n* = 190) were detected or confirmed using our recently implemented RT-qPCR in the Panther Fusion^®^ System. During these initial weeks, we tested NP samples from 4689 individuals, 58.9% female and most of them (93.1%) between 15 and 75 years old (median 41 years, 95% CI: 41–42 years). A total of 190 individuals (4%) were positive for SARS-CoV-2, 52.6% female and the majority (85.8%) ranging in age between 20 to 30 and 45 to 60 years old (median 37 years, 95% CI: 37–44 years, Figure 4B). Out of 375 NP samples tested for SARS-CoV-2 that were also tested for other respiratory viral pathogens (AusDiagnostics Respiratory Pathogens C Assay, AusDiagnostics NZ Ltd., Auckland, New Zealand), 313 (83.5%) were negative not only for SARS-CoV-2 but for six other respiratory viruses (Figure 4C). Fifty-four of the SARS-CoV-2 negative samples were positive for Influenza A virus (*n* = 37), Rhinovirus/Enterovirus (*n* = 7), Respiratory Syncytial Virus (*n* = 6), Influenza B virus (*n* = 3), and/or Parainfluenza virus 3 (*n* = 1). Interestingly, none of the eight NP specimens positive for SARS-CoV-2 in this batch of 375 samples were positive for any other of the six respiratory viruses (Figure 4C).

### 3.4. 17 to 20 March 2020: Characterizing the First Two SARS-CoV-2 Infections in the South Island, New Zealand

Once we used the recently implemented RT-qPCR assay to identify the first COVID-19 case in the South Island, we confirmed the diagnosis by sequencing the whole genome of the virus. For that, we used a metagenomics approach to randomly construct cDNA from RNA extracted from the NP sample, which was promptly sequenced in the MinION platform (Oxford Nanopore Technologies). Patient-derived sample NZ/Queenstown/01 generated a total of 322,241 quality reads, with an average sequence length of 594 bp and an average quality score of 9.7. Interestingly, sequences from the genus *Betacoronavirus* were at the top (33,720 cumulative reads, 10.5% of the total reads analyzed) of the 10 most abundant sequences identified using the EPI2ME real-time data analysis platform (Oxford Nanopore Technologies), with the other nine corresponding to bacteria and fungi sequences from genera, such as *Escherichia*, *Malassezia*, and *Prevotella*.

We then decided to confirm the results obtained with the MinION system (Oxford Nanopore Technologies) by re-sequencing the sample from the first patient (NZ/Queenstown/01), together with the first COVID-19 case identified in Dunedin (NZ/Dunedin/01), on the MiSeq platform (Illumina). A total of 2,032,274 reads passing filter were obtained from the single MiSeq v2 Nano run, with 1071 cluster density, 0.47% error rate, 71% clusters passing filter, and 88% QScore ≥ Q30. Reads were mapped, and whole genomes were assembled de novo or using a reference template (SARS-CoV-2 isolate Wuhan-Hu-1 NC_045512) depending on the software tool used. As expected, all four analyses (GISAID, DRAGEN Bio-IT Platform, Genome Detective Virus Tool, and IDSEQ) generated similar results for both NZ/Queenstown/01 and NZ/Dunedin/01 samples: total mapped reads (867,404 and 873,057 reads), median coverage depth (220 and 55 reads), and contig length (27,729 and 29,685 bp). In fact, the whole genome SARS-CoV-2 sequences generated using the different software analyses were separated into two patient-specific phylogenetic clusters supported by 100% bootstrap values (Supplementary Figure S1). Interestingly, and confirming the findings observed with the MinION (Oxford Nanopore Technologies), the metagenomics approach used with the MiSeq (Illumina) and analyzed with IDSEQ also identified sequences from a series of bacterial genera, such as *Escherichia*, *Streptococcus*, *Veillonella*, *Prevotella*, and *Staphylococcus*, most likely associated with the nasopharynx environment of the patients.

**Figure 5:**
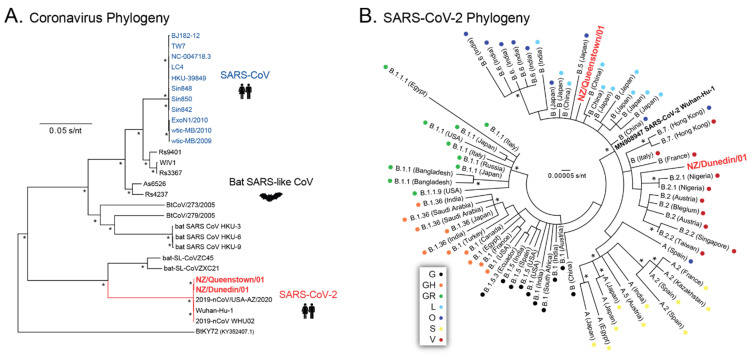
Phylogenetic analysis of the first two SARS-CoV-2 infections in the South Island, New Zealand. Maximum Likelihood phylogenetic trees were constructed using whole-genome SARS-CoV-2 consensus sequences corresponding to the first two COVID-19 cases identified in the South Island (NZ/Queenstown/01 and NZ/Dunedin/01) and 28 SARS-like betacoronaviruses (**A**) or 70 contemporary SARS-CoV-2 sequences from different lineages (**B**) obtained from GISAID database (https://www.gisaid.org/, accessed on 15 April 2020) in June 2020. Each color-coded dot represents SARS-CoV-2 GISAID clades. Bootstrap resampling (1000 data sets) of the multiple alignments tested the statistical robustness of the trees, with percentage values above 75% indicated by an asterisk. s/nt, substitutions per nucleotide.

To characterize the SARS-CoV-2 infecting the first two cases identified in the South Island (NZ/Queenstown/01 and NZ/Dunedin/01), we first reconstructed the phylogeny of the two new SARS-CoV-2 sequences using 28 SARS-like betacoronavirus sequences downloaded from the GISAID database (https://www.gisaid.org/, accessed on 15 April 2020), which clearly showed that both patient-derived viral sequences clustered with other SARS-CoV-2 sequences (Figure 5A). Further phylogenetic analysis using 70 contemporary SARS-CoV-2 sequences from different lineages, as well as blast comparisons using a series of databases, i.e., Nextstrain (https://nextstrain.org/ncov/, accessed on 15 April 2020), PANGO Lineages (https://cov-lineages.org/index.html, accessed on 15 April 2020), Los Alamos COVID-19 Viral Genome Analysis Pipeline (https://cov.lanl.gov/content/index, accessed on 15 April 2020), and COVID-19 Research at UCSC (https://hgwdev.gi.ucsc.edu/covid19.html, accessed on 15 April 2020), classified these two new SARS-CoV-2 sequences as: Nextstrain Clade 19A, Pango Lineage B.55, and GISAID clade B(*L*) for NZ/Queenstown/01 and Nextstrain Clade 19A, Pango Lineage B.31, and GISAID clade B.2(*O*) for NZ/Dunedin/01 (Figure 5B).

**Figure 6:**
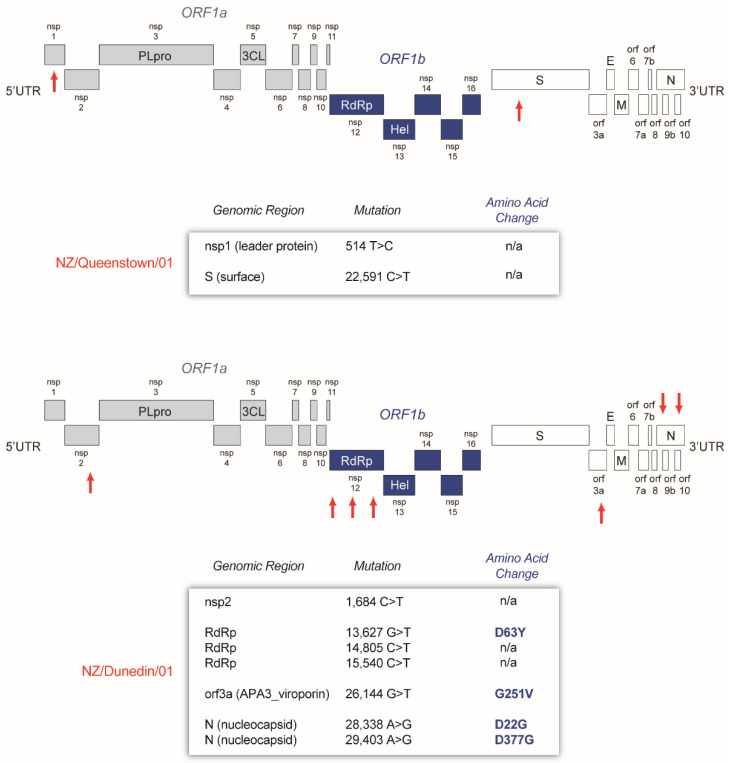
SARS-CoV-2 genome structure depicting the single nucleotide polymorphisms (red arrows) in both NZ/Queenstown/01 and NZ/Dunedin/01 SARS-CoV-2 sequences relative to the Wuhan-Hu-1 (NC_045512) SARS-CoV-2 reference strain. n/a, not applicable (synonymous mutation).

Finally, we were able to identify a few single nucleotide polymorphisms (SNP or mutations) in the new SARS-CoV-2 sequences from New Zealand, relative to the reference sequence (SARS-CoV-2 isolate Wuhan-Hu-1 NC_045512). NZ/Queenstown/01 had only two synonymous mutations in the nsp1 (leader protein) and the spike genes, while NZ/Dunedin/01 had seven SNPs, including four nonsynonymous mutations in the RdRp (D63Y), orf3a (G251V), and the nucleocapsid (D22G and D377G, Figure 6).

## 4. Discussion

The COVID-19 pandemic has taken the world by storm. Since the first cases of viral pneumonia in Wuhan, China, in December 2019 [1,7], SARS-CoV-2 has caused havoc around the planet, affecting not only global health but also the world economy [25]. In a matter of months, if not weeks, academic, clinical, and public health laboratories had to race to develop and distribute reliable diagnostic tests for the new human coronavirus. Here, we describe our experience (i) implementing a molecular test to detect SARS-CoV-2 infection, (ii) adapting the RT-qPCR assay to be used in a random-access platform (Hologic Panther Fusion^®^ System) in a clinical laboratory, and (iii) characterizing the first whole-genome SARS-CoV-2 sequences in the South Island, at the onset of the SARS-CoV-2 outbreak in New Zealand.

There is no doubt that the rapid sequencing and publication of the whole genome of 2019-nCoV (SARS-CoV-2) in January 2020 [1] facilitated the development of a variety of assays to diagnose patients infected with this new coronavirus. In approximately 18 months, hundreds—if not thousands—of in vitro diagnostic (IVD) devices have been developed [11,14], some of them approved by regulatory agencies, such as the US Food and Drug Administration (FDA) and/or the European Medicine Agency (EMA). In fact, as of 26 August 2020, the FDA had granted emergency use authorization for 255 molecular and 33 antigen diagnostic tests for SARS-CoV-2, 87 serology, and other adaptive immune response tests for SARS-CoV-2, and four tests for the management of COVID-19 patients (https://www.fda.gov, accessed on 20 August 2021). Unfortunately, that was not the case in early 2020, when just a handful of protocols and laboratory-developed tests (LDT) were available. It was during the last week of January 2020 that we decided to implement one of these assays in Dunedin, New Zealand, anticipating the possibility that the new coronavirus disease could reach our shores.

The Drosten group from the Charité Universitätsmedizin Berlin Institute of Virology in Germany was one of the first research laboratories to develop a robust diagnostic assay for SARS-CoV-2 infection [8]. On 17 January 2020, they released a detailed workflow protocol online for detecting 2019-nCoV using real-time RT-PCR. We quickly adapted their assay, ordering primers and probes as described in their protocol, as well as plasmids containing fragments of the SARS-CoV-2 E and RdRp genes to be used as controls. Like many other research laboratories around the world, we verified the specificity and analytical sensitivity of the RT-qPCR assay using in vitro-transcribed RNA from DNA plasmids containing synthetically generated SARS-CoV-2 gene fragments. We then decided to adapt the molecular test for use on the Panther Fusion^®^ System (Hologic), a fully automated diagnostic platform [19]. Although today there is a validated test specially designed to work on this instrument, i.e., the Panther Fusion SARS-CoV-2 Assay (Hologic) [12,26,27], that was not the case in February 2020, at least not in New Zealand. The Panther Fusion^®^ System is a random access, sample-to-answer diagnostic platform capable of isolating genetic material and performing internally-controlled multiplex RT-PCR [19]. We took advantage of the Open Access function of the instrument to implement the “Drosten protocol” in this fully automated platform. We focused on detecting the SARS-CoV-2 E gene as a diagnostic method, using the RdRp gene as a confirmatory test. Corman et al. [8] reported highly sensitive assays based on these two viral genes, i.e., detecting 5.2 and 3.8 RNA copies/reaction for the E and RdRp genes, respectively. In our hands, using the Panther Fusion^®^ System (Hologic), the LOD was 30 and 300 RNA copies/reaction of the E and RdRp genes, respectively. The 6- to 100-fold difference in assay sensitivity could be attributed to differences in sample types, RNA extraction methods, and/or any other intrinsic characteristic of the different RT-qPCR platforms used. It would be interesting to compare our version of the Drosten assays adapted to run in the Panther Fusion^®^ System with the current Panther Fusion^®^ SARS-CoV-2 Assay (Hologic), which has been shown to have a LOD ranging from 74 to 500 RNA copies/mL [28,29]; however, this was not only beyond the scope of this initial study but impossible to perform during the early stages of the COVID-19 pandemic in New Zealand.

It was during the week of 13 March 2020, when our newly implemented SARS-CoV-2 assay was ready in the clinical laboratory, that the first few cases of COVID-19 were identified in the South Island, New Zealand: the ideal debut and test for our assay. At the time, clinical laboratories across the country were using other LDTs to diagnose SARS-CoV-2 infections [30,31]. We showed that our assay performed similarly to the other contemporary molecular diagnostic tests, correctly identifying (or not) infections by SARS-CoV-2. To this date, the assay continues to be performed in the Southern Community Laboratories, Dunedin Hospital, although mainly as a backup test to several commercial assays, such as the Panther Fusion^®^ SARS-CoV-2 Assay (Hologic), the Aptima^®^ SARS-CoV-2 Assay (Hologic), and the Tapath COVID-19 Combo Assay (ThermoFisher Scientific).

As of 31 August 2021, more than 3 million SARS-CoV-2 sequences had been submitted to several databases, including GISAID (https://www.gisaid.org/, accessed on 31 August 2021), one of the leading organizations tracking the virus responsible for the COVID-19 pandemic. This massive amount of information has been extremely valuable in understanding the evolution and epidemiology of the virus [7,32,33], leading to the classification of SARS-CoV-2 genetic diversity into clades or lineages [34]. Nine larger clades have been described in GISAID, 19 clades by Nextstrain (https://nextstrain.org/ncov/, accessed on 31 August 2021), while an increasing number of PANGO lineages (https://cov-lineages.org, accessed on 31 August 2021) are used to track the transmission and spread of SARS-CoV-2 worldwide, including several variants of interest or concern [35]. The SARS-CoV-2 sequences described here were obtained very early in the pandemic; thus, they were epidemiologically close to the original SARS-CoV-2 sequences described in Wuhan, China [1]. The two individuals, from Queenstown and Dunedin, were confirmed on 17 March 2020 as the first COVID-19 cases identified in the South Island. The rapid sequencing of the first whole SARS-CoV-2 genome from Queenstown, NZ (NZ/Queenstown/01) using the MinION system (Oxford Nanopore Technologies) was confirmed two days later, together with the first whole SARS-CoV-2 genome from Dunedin, NZ (NZ/Dunedin/01), using the MiSeq platform (Illumina). Phylogenetic analyses using a variety of approaches and software tools available both online and in our laboratory confirmed that both viral sequences corresponded to SARS-CoV-2. Further analysis classified the two new SARS-CoV-2 sequences as 19A/B.55/B(*L*) and 19A/B.31/B.2(*O*) based on Nextrain Clade/Pango Lineage/GISAID Clade for NZ/Queenstown/01 and NZ/Dunedin/01, respectively. Interestingly, SARS-CoV-2 sequences from the Pango lineage B.55 (reassigned from lineage B.11, first identified in January 2020) were associated with viruses from Europe, including the Netherlands, United Kingdom, and Germany (https://cov-lineages.org, accessed on 31 August 2021). The patient from Queenstown (NZ/Queenstown/01) had returned from Germany before being diagnosed with COVID-19. On the other hand, the SARS-CoV-2 sequence from the Dunedin patient (NZ/Dunedin/01) was classified as Pango lineage B.31 (reassigned from lineage B.2.5, initially identified in March 2020), which has been associated with viruses from the United States, New Zealand, and Australia (https://cov-lineages.org, accessed on 31 August 2021). A comprehensive analysis of over 600 SARS-CoV-2 genomes obtained from patient-derived samples taken between February and May 2020 in New Zealand showed a high degree of viral diversity, including multiple A and B lineages, mostly B.1, B.1.1., and B.1.26, with a low frequency of B.2.5 (B.31) [36]. Other variants have been introduced, such as B.1.1.1 (the now extinct C.12 lineage) associated with the second SARS-CoV-2 outbreak that occurred in August 2020 [37], while viruses from other lineages have been imported but restricted to the quarantine facilities over the course of the pandemic, e.g., B.1.509 [18]. Nevertheless, the worldwide cumulative prevalence of the SARS-CoV-2 lineages described in this study, from early March 2020 (B.55 and B.31), has diminished considerably, to less than 0.5%, since April 2020 (https://cov-lineages.org, accessed on 31 August 2021).

We identified only nine SNPs relative to the reference sequence (SARS-CoV-2 isolate Wuhan-Hu-1 NC_045512) in the first two SARS-CoV-2 sequenced in the South Island, NZ, in March 2020. The virus from the first patient (NZ/Queenstown/01) showed just two synonymous mutations, highlighting the close relationship with the original viral variants, most likely spreading to Germany from China, then to New Zealand during the early phase of the pandemic. The second SARS-CoV-2 sequence (NZ/Dunedin/01) showed seven mutations, including four amino acid substitutions in the RdRp, orf3A, and nucleocapsid. In fact, two of these mutations (G251V in the orf3a and D22G in the nucleocapsid) correspond to signature polymorphisms associated with the B.31 lineage [34,35]. To our knowledge, none of the mutations described in these two patient-derived sequences have been associated with changes in viral phenotype.

SARS-CoV-2 arrived late in 2019, and the world was not ready for it. Despite an incredible effort by researchers, clinicians, and public health officers around the world, the virus was able to spread and is responsible for one of the longest and more deadly pandemics of the last 100 years. Here we describe our experience implementing, deploying, and validating a diagnostic test in advance of the potential arrival of an emergent virus to New Zealand. We were also able to confirm the first COVID-19 cases in the South Island by sequencing and characterizing the first whole-genome SARS-CoV-2 sequences in the Southern DHB. Furthermore, this seminal work led to the first isolation of SARS-CoV-2 in New Zealand (R. Harfoot, B. Lawley, and M.E. Quiñones-Mateu, Characterization of the first SARS-CoV-2 isolates from Aotearoa New Zealand. *Viruses*, submitted for publication), a critical step towards numerous academic, clinical, and commercial projects in the country. We are confident that our work will help us and others prepare for the unequivocal risk of similar viral outbreaks in the future.

## Data Availability

Raw data can be requested by contacting the corresponding authors.

## Supplementary Materials

The following are available online at https://www.mdpi.com/article/10.3390/v13112222/s1, Figure S1: Coverage and phylogenetic analysis using four different software packages.
