# Supplementary material for: Rapid Response to SARS-CoV-2 in Aotearoa New Zealand: Implementation of a Diagnostic Test and Characterization of the First COVID-19 Cases in the South Island
Source: Viruses. 2021 Nov 4;13(11):2222. doi: 10.3390/v13112222 (PMC8623489; doi:10.3390/v13112222)
Supplement: Supplementary file 1 [file viruses-13-02222-s001.zip › viruses-1394394-supplementary.pdf]

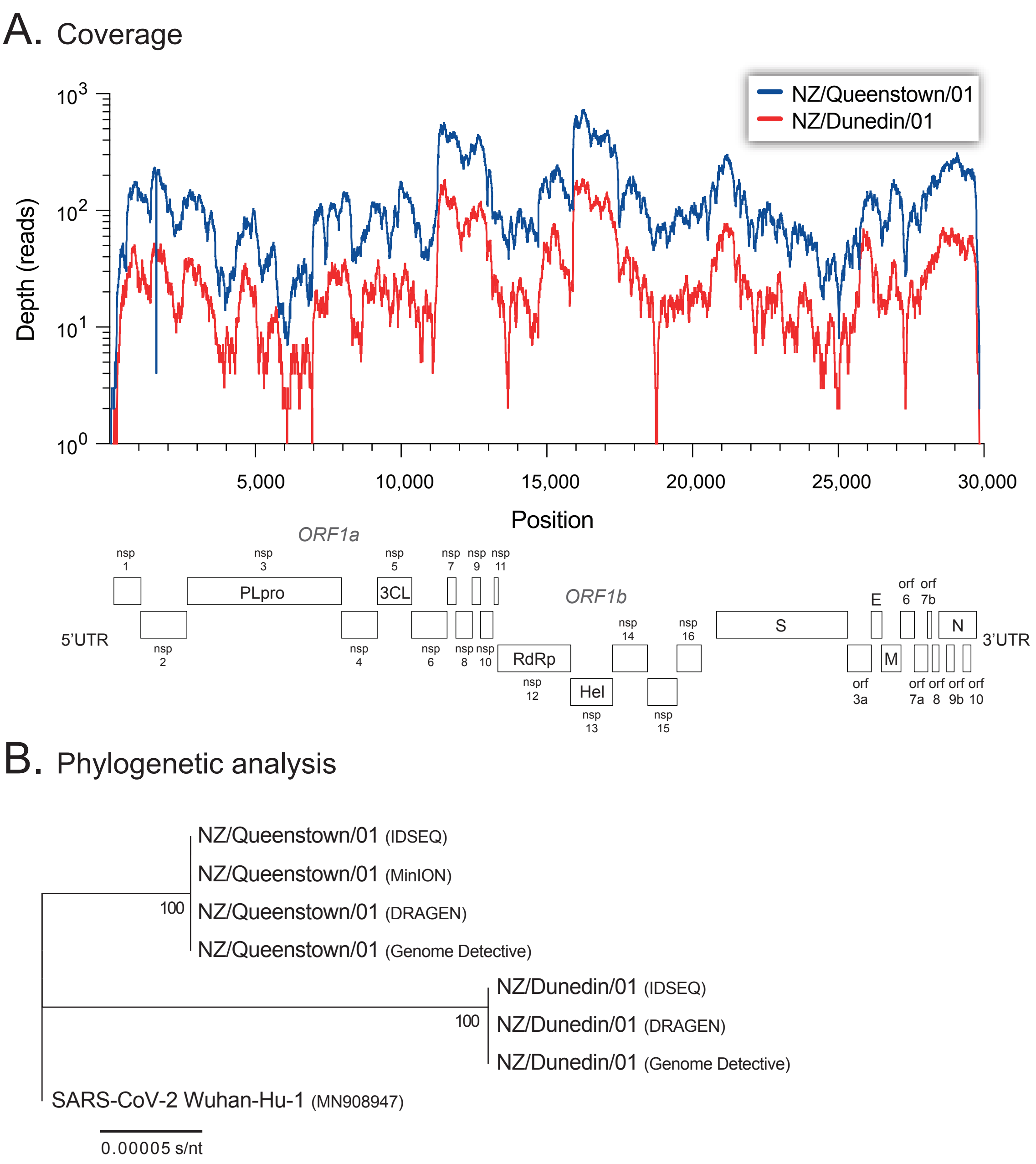

Supplementary Figure S1. (A) Coverage, i.e., number of reads per nucleotide position, obtained by deep sequencing of the first two COVID-19 cases identified in South Island, New Zealand. Near whole genome SARS-CoV-2 sequences were obtained as described in Materials and Methods. The position relative to the Wuhan-Hu-1 (NC_045512) SARS-CoV-2 reference strain is indicated. (B) Maximum Likelihood phylogenetic tree constructed using whole genome SARS-CoV-2 sequences generated (mapped and aligned) using four different software tools: GISAID, DRAGEN Bio-IT Platform (Illumina), Genome Detective Virus Tool, and IDSEQ (see Materials & Methods for details. Bootstrap resampling (1,000 data sets) of the multiple alignments testing the statistical robustness of the trees (%) is indicated. s/nt, substitutions per nucleotide.
